# Homozygosity for Mobile Element Insertions Associated with *WBSCR17* Could Predict Success in Assistance Dog Training Programs

**DOI:** 10.3390/genes10060439

**Published:** 2019-06-09

**Authors:** Dhriti Tandon, Kyra Ressler, Daniel Petticord, Andrea Papa, Juliana Jiranek, Riley Wilkinson, Rebecca Y. Kartzinel, Elaine A. Ostrander, Nathaniel Burney, Carol Borden, Monique A. R. Udell, Bridgett M. VonHoldt

**Affiliations:** 1 Department of Ecology & Evolutionary Biology, Princeton University, Princeton, NJ 08544, USA; dp10@princeton.edu (D.P.); larkin.papa49@gmail.com (A.P.); jjiranek@princeton.edu (J.J.); rileyw@princeton.edu (R.W.); 2 Mercer County Community College, West Windsor, NJ 08550, USA; kyraress@gmail.com; 3 Department of Ecology & Evolutionary Biology, Brown University, Providence, RI 02912, USA; rebecca_kartzinel@brown.edu; 4 Cancer Genetics and Comparative Genomics Branch, National Human Genome Research Institute, National Institutes of Health, Bethesda, MD 20892, USA; eostrand@mail.nih.gov; 5 Guardian Angels Medical Service Dogs, Inc., Williston, FL 32696, USA; nburney@medicalservicesdogs.com (N.B.); carol@medicalservicedogs.com (C.B.); 6 Department of Animal and Rangeland Sciences, Oregon State University, Corvallis, OR 97331, USA; monique.udell@oregonstate.edu

**Keywords:** *Canis*, genetics, transposons, hypersociability, domestication

## Abstract

Assistance dog training programs can see as many as 60% of their trainees dismissed. Many training programs utilize behavioral assays prior to admittance to identify likely successful candidates, yet such assays can be insconsistent. Recently, four canine retrotransposon mobile element insertions (MEIs) in or near genes *WBSCR17* (Cfa6.6 and Cfa6.7), *GTF2I* (Cfa6.66) and *POM121* (Cfa6.83) were identified in domestic dogs and gray wolves. Variations in these MEIs were significantly associated with a heightened propensity to initiate prolonged social contact or hypersociability. Using our dataset of 837 dogs, 228 of which had paired survey-based behavioral data, we discovered that one of the insertions in *WBSCR17* is the most important predictor of dog sociable behaviors related to human proximity, measured by the Canine Behavioral Assessment Research Questionnaire (C-BARQ©). We found a positive correlation between insertions at Cfa6.6 and dog separation distress in the form of restlessness when about to be left alone by the owner. Lastly, assistance dogs showed significant heterozygosity deficiency at locus Cfa6.6 and higher frequency of insertions at Cfa6.6 and Cfa6.7. We suggest that training programs could utilize this genetic survey to screen for MEIs at *WBSCR17* to identify dogs with sociable traits compatible with successful assistance dog performance.

## 1. Introduction

Genome-wide approaches have increased in their application for exploring the molecular basis of animal behavior and personality. In contrast to many dog morphological traits, which have been successfully mapped to genetic variants [1,2,3], efforts to understand the causal genetic variants of dog temperament and personality are relatively new. Such insights can have positive implications for decisions regarding animal health and welfare. Although behavioral assays are currently used to identify extreme behavioral conditions in dogs, these methods are sensitive to the testing environment (e.g., stress behaviors are often documented in recently captured or relinquished shelter dogs) [4] and may not be highly predictive of long-term positive behaviors [5,6]. Additionally, as dog personality varies with age [7], behavioral assays likely face reduced accuracy for predicting adult behavior when conducted during sub-adult developmental stages [8,9,10]. These limitations are compounded by the reliance of matching behavioral assays with the targeted dog behavioral traits, often with behavioral observations recorded by a non-specialist who has spent limited time with the study subject [11,12,13]. Consequently, there is an immediate need for the development of an accurate, unbiased behavioral assessment technique. The Canine Behavioral Assessment Research Questionnaire (C-BARQ©) is a popular and validated questionnaire-based behavioral assessment tool used by pet owners, breeders, and trainers to evaluate their dogs. The C-BARQ© describes dog behavior with respect to temperament, fear, trainability, and anxiety [14]. Here, we propose the combinatorial addition of a genetic assay that is easy to conduct alongside the C-BARQ©, which could improve the prediction of heritable behavior in adult dogs. The combination of a questionnaire and genetic assay would reduce the time and effort required of other behavioral assessments, an aspect that may be especially beneficial when evaluating assistance dog candidates for entry into their respective time- and cost-intensive training programs.

Assistance dogs are tremendously valuable with respect to their owner’s physical and mental wellbeing [15,16]. These include guide dogs (those that assist people with visual disabilities), hearing dogs (those that assist people with hearing impairments), and service dogs (those that assist people with motor disabilities) [16,17]. Assistance dog training programs explicitly survey behaviors that are imperative to their program’s success, and include robustness to environmental stressors, social behaviors, trainability, attention, and problem-solving [6]. However, these programs often document up to 60% failure rates [18], with behavioral problems reported as one of the primary causes for dismissal. For instance, 60% of dogs that are withdrawn from the Guide Dogs for the Blind program for behavioral reasons that include high activity level, incompatibility with cats or other dogs, and assertiveness toward leadership [19]. Studies have identified that human-directed sociability behaviors, such as docility [20] and fear towards strangers [6], have a profound effect in predicting success at assistance dog training programs. Additionally, dogs that fail assistance dog training programs often score significantly lower on C-BARQ© behaviors that correspond with human-directed sociability [21]. While the accuracy of behavioral assessment tools ranges from 64% and 87%, large variability exists in the positive predative value of assistance dog performance (8.4–93.3%), a measure that quantifies the likelihood that dogs classified as unsuccessful will be subsequently dismissed from training programs [5]. An age-independent genetic assay that is informative for specific sociability-related behavioral traits could aid in efforts to identify candidates with social profiles that are well-suited for an assistance role. This information could influence training or placement decisions early in the dog’s development, something that could provide welfare, economic benefits, and positive impact on public health.

Recently, we discovered four canine retrotransposon mobile element insertions (MEIs) in domestic dogs and gray wolves that were significantly associated with a heightened propensity to initiate prolonged social contact, commonly referred to as hypersociability [22]. These MEIs are located on canine chromosome 6 (CFA6) and associated with the genes *WBSCR17* (Cfa6.6 and Cfa6.7), *GTF2I* (Cfa6.66), and *POM121* (Cfa6.83), which are known to be involved in Williams–Beuren syndrome in humans [23]. VonHoldt et al. found that MEIs within the genes *WBSCR17* and *POM121* were associated with increased hypersociability, while MEIs within the gene *GTF2I* were associated with decreased hypersociability [22]. Additionally, higher copy number in *WBSCR17* and *POM121* were associated with increased breed groups by their stereotyped attention-seeking behaviors [22]. These MEIs were also found to segregate within breeds as well as wolves, and significantly associated with changes in social behavior after accounting for species membership [22]. These MEIs are retrotransposon elements [22,24], and hence are highly methylated [25]. Through a survey of methylation and transcriptional data, these MEIs were found to affect gene expression likely via *cis*-regulatory pathways. MEIs are highly methylated and may regulate adjacent genes [24]. These elements are easy to genotype through a targeted PCR protocol, and thus identifying MEI copy number at these four loci is straightforward.

Here, we assessed patterns of MEI insertions of dogs with varying sociable personalities and explored the frequency of MEIs across various dog groups (e.g., assistance dogs, breeds). The C-BARQ© assesses dog attachment seeking, which is described by an individual’s propensity to seek physical proximity to owners and heightened anxiety when separated from owners [14]. In this study, we determined the association between MEI copy number and behavioral scores on the C-BARQ©, and whether MEI genotypes can predict attachment style. Secondly, we elucidated differences in C-BARQ© behaviors and MEI copy number between assistance and pet dogs, as well as copy number differences between breed groups. 

## 2. Materials and Methods

All dog owners gave their informed consent for inclusion and participation of their dogs in the study. The study was carried out under the IACUC protocol number 2098 A-17.

### 2.1. Genotyping Four Mobile Element Insertions Associated with Human-Directed Hypersociability

We isolated DNA from 837 adult domestic dogs >1 year of age (Table S1), 159 of which were whole blood samples and 678 from buccal cells or saliva using Qiagen’s DNeasy Blood and Tissue Kit (Qiagen, Germantown, MD, USA). Our samples were derived across 74 breeds (*n*: purebred = 656, mixed-breed = 104, unknown = 78), from 196 assistance dogs (sample size per breed: German Shepherds = 56, Golden Retriever = 29, Labrador Retriever = 118) and 642 pet dogs. For all DNA isolated from buccal cells or saliva, we completed a second purification step using a 1:2 ratio of DNA to AMPure XP magnetic purification beads (Beckman Coulter Life Sciences, Indianapolis, IN, USA). We followed previously published amplification methods to genotype and survey the insertional dynamics of four MEIs implicated in canine hypersocial behavior [22]. We obtained amplicons between 215 bp and 555 bp in length, and the total PCR product was visualized on a 1.8% agarose gel for scoring genotypes as the number of insertions per locus per individual (0, 1, or 2) (Tables S2 and S3). To note, our study included 56 German Shepherd assistance dogs (juveniles of <1 year = 7; adults of 1–5 years = 49) from Guardian Angels Medical Service Dogs, Inc., a facility whose mission is to rescue, raise, and train medical and assistance dogs. The study also included 118 Labrador Retriever and 29 Golden Retriever guide dogs from Guide Dogs for the Blind (GDB), an organization started in the 1940s in San Rafael, California.

### 2.2. Canine Questionnaire Data to Identify Behavioral Types

Of 837 dogs with genetic samples, 228 also had paired detailed demographic (age, sex, breed, DOB, and # years owned) and behavioral data derived from 42 questions of the C-BARQ© (short version) [14]. The C-BARQ© quantifies the behavioral tendencies of individual dogs (as assessed by an owner, handler, or evaluator) across 14 behavioral categories by averaging scores across related questions (Table S4). Questions from the attachment, attention-seeking, and separation distress sections of the evaluation most closely parallel behavioral traits used to identify dogs displaying hypersociability (elevated proximity-seeking) in prior research (Table S5). Additionally, stranger-directed aggression and stranger-directed fear quantify opposing behaviors towards unfamiliar people, and hence would be negatively correlated with prosocial interest in strangers (Table S5). Questions from the trainability and chasing sections (questions Q29 and Q30, respectively) closely match the attention bias to stimuli behavioral summary; however, the C-BARQ© questionnaire does not specify the nature of the stimulus in terms of being social or nonsocial [14] (Table S5).

Previous studies have shown that dog behavior varies with age. We conducted Spearman’s rank correlational tests to elucidate associations between dog age and sociability measures on the C-BARQ©. We also created four datasets of pet dogs based on demographics from the C-BARQ© to ensure that we accounted for differences in behavior that may be due to the age of the dog or how many years the dog was owned at the time of reporting. Each of these datasets has paired MEI genotype and behavioral C-BARQ© data. The datasets comprising of pet dogs were as follows: (1) 69 young adult dogs of 1–5 years of age; (2) 95 adult dogs over >5 years of age; (3) 65 dogs that have been owned for between 1 and5 years by the reporting owner; and (4) 98 dogs that have been owned for >5 years by the reporting owner (Table S1). Additionally, our dataset included 49 German Shephard assistance dogs, all of 1–5 years of age, with paired genotype and C-BARQ© data. Some samples lacked data regarding the number of years a dog had been owned and date of birth. These samples were excluded from all subsequent analyses, whereby age is a relevant stratification factor.

### 2.3. Predictive Power of the Mobile Element Insertion’s Copy Number

To elucidate the potential order of importance of these loci in affecting sociability-related phenotypes, we employed conditional random forests ensemble algorithms using 20,000 bootstrap samples from the R package “party” [26] and function “party” for all dogs of 1–5 years of age. C-BARQ© behavioral axes related to attachment-seeking and separation distress behaviors were used as response variables. Conditional random forests were used to alleviate confounding importance measures due to interdependency of the loci [27,28]. Variable importance measures (VARIMP) were computed using the function “varimp”, setting the conditional parameter to “true” [26]. Variables with a positive VARIMP increase predictive power, while variables with a negative VARIMP decrease predictive power [29]. A large positive VARIMP indicates a potentially predictive variable [29]. We analyzed two types of models: (1) the inclusion of all loci, with each as a predictor; and (2) the exclusion of locus Cfa6.66 due to its low interindividual variation. 

### 2.4. Mobile Element Insertion Copy Number and Correlation with C-BARQ© behaviors

Previously, hypersocial canine behavior was quantified using solvable tasks and sociability measures, including dogs’ propensity to spend time looking at a human relative to a nonsocial stimulus (referred to as attentional bias) and their propensity to spend time in proximity to familiar or unfamiliar humans (hypersociability and social interest in strangers, respectively) [22]. To determine which personality axes of the C-BARQ© are predicted by the MEI genetic test, we first constructed four datasets of pet dogs to control for age and the number of years the dog had been owned. We created two additional datasets which include both pet and assistance dogs for dogs of age 1–5 years (*n* = 117) and those owned from 1–5 years (*n* = 115), since all assistance dogs containing C-BARQ© scores belonged to these groups. To assess whether relevant questions on the C-BARQ© tag the same behaviors as tested in vonHoldt et al. [22], we modeled the associations between MEI copy number and the C-BARQ© questions hypothesized to be informative for social behavior. We measured the associations between MEI copy number and C-BARQ© scores using linear ridge regression [30]. Ridge regression considers the correlation between predictor variables and corrects for multicollinearity [30]. We analyzed the datasets using the R package “ridge” [31] and implemented identical parameter settings for each dataset to control for age and number of years owned. Age and sex were included as covariates in the models. Beta values and p-values were estimated for each relevant question and for the average scores across all questions tagging the same general behaviors as per vonHoldt and colleagues [22], which include hypersociability (average of Q22, Q23, Q24, Q25, and Q26), social interest in strangers (average of Q3, Q9, Q13, and Q15), and attention bias to social stimuli (average across Q29 and Q32). Higher averages on Q3, Q9, Q13, Q15 and Q29, Q32 correspond to lower social interest in strangers and attention bias to stimuli, respectively.

### 2.5. Comparing Mobile Element Insertion of Assistance and Pet Dogs

We assessed differences in C-BARQ© behavioral scores and MEI copy number comparing assistance and pet dogs. Differences in C-BARQ© behavior and MEIs were assessed using Mann–Whitney U tests with a Bonferroni correction implemented with the R functions wilcox.test and “p.adjust” [32]. To assess whether each group experienced heterozygosity deficiency at a locus, we estimated within group differences in heterozygosity (observed, H_O_) and allele frequencies with the Hardy–Weinberg equilibrium (HWE) exact test function “Hwe.exact” in the R “genetics” package [33]. Between-group differences were analyzed using the Bonferroni-corrected Fisher’s exact test (“fisher.exact”) [32]. Paired C-BARQ© scores and genotype data were present for assistance dogs belonging to a single breed, the German Shepherd. Consequently, we repeated the analysis for genotypic differences of pure-bred dogs belonging to a single breed clade called Retrievers to ensure, at a certain extent, that genotypic differences detected in the previous analysis are not breed-specific and instead truly represent differences between assistance and pet dogs, regardless of breed.

### 2.6. Rationale for Breed Group Categories

We hypothesize that the MEIs will display allele frequency differences for breeds with respect to their genetic relationships with other breeds and their own unique breed history. We therefore grouped dogs into breed groups based on previous studies that identified the genetic relationships between breeds using phylogenetic trees. Several studies have reported breed groups that are highly genetically divergent and distinct. The primary signals of divergence are from Asian Spitz-type breeds, Arctic Spits-type breeds, Sighthounds, and African breeds, which branched separately from European-derived breeds [34,35,36]. Many of these aforementioned breed groups have histories that consider them ancient in origins compared to those known to have a more recent establishment with subsequent rapid radiation, although both breed groups have experienced augmentation throughout their existence [37,38,39]. Following these previously studies, we have grouped these genetically distinct breeds in a category called divergent and the remainder of breeds into the recent-radiation category.

### 2.7. Comparing Mobile Element Insertions in Divergent and Recent-Radiation Breeds

We assessed MEI insertion frequency in dogs belonging to either divergent or recent-radiation breed groups. We hypothesized that selection for human-directed hypersociability pre-Victorian-era breed radiation likely resulted in differences in sociability and MEI frequencies before and after the Victorian breed radiation [37,39]. As the recent radiation rapidly developed numerous new breeds with targeted functions, we suspect that many breeds were not selected explicitly for hypersociability and the associated MEIs likely drifted or were purged. We categorized dog breeds regarding their documented origins (n, divergent = 52, recent radiation = 324) following previously published classifications [34,35] (Table S6). Within-group genotype and allele frequency differences were assessed using the Hardy–Weinberg equilibrium (HWE) exact test using the function “Hwe.exact” on the R package “genetics” [33]. Between-group allele frequency differences were analyzed using the Bonferroni-corrected Fisher’s exact test (“fisher.exact”) [32]. Assistance dogs were excluded from these analyses. As divergent breeds represented a smaller fraction of our dataset (*n* = 52), we randomly sampled 100 individuals from the recent-radiation group to ensure sample size differences did not introduce any biases. Only pure-bred dogs were used for this analysis.

## 3. Results

### 3.1. Dog Sociability Trends May Shift with Age

Using Spearman correlational tests to evaluate age based trends in the C-BARQ© behavioral axes, we found that on average, an increase in age (age range: 1–17 years; median age: 10 years) corresponded to a decrease in attachment and attention-seeking behavior (*R* = −0.14, *p* = 0.03), a decrease in separation-related problems (*R* = −0.23, *p* ≤ 0.001), and an increase in stranger-directed aggression (*R* = 0.34, *p* ≤ 0.001) (Figure S1).

### 3.2. Cfa6.6 Has the Highest Predictive Power of Sociability-Related Behaviors for Dogs Aged 1–5 Years

**Figure 1:**
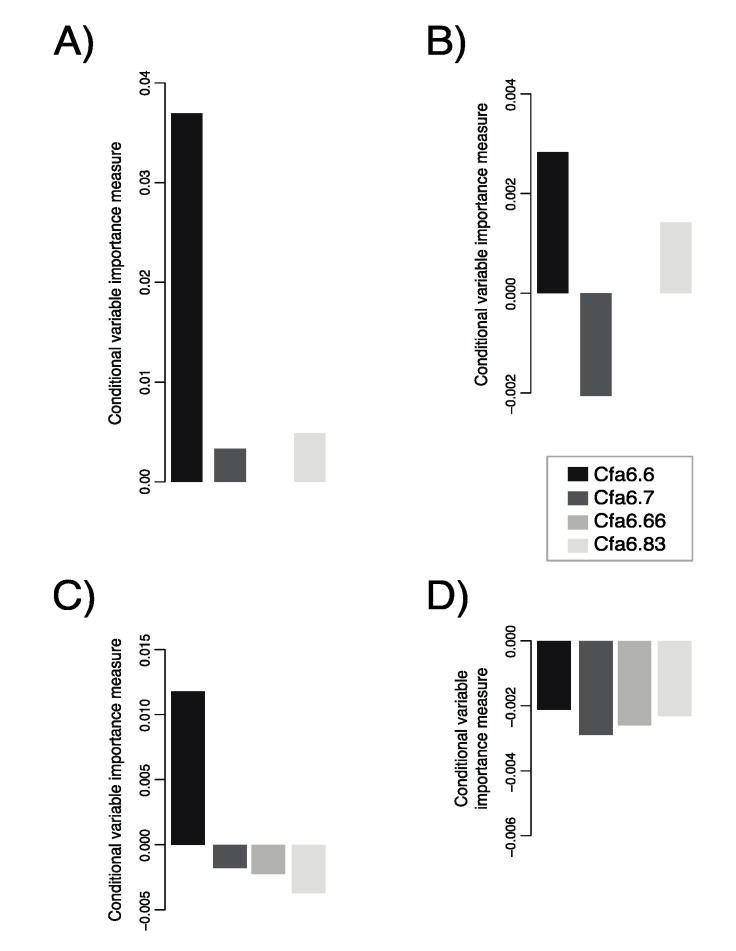
The variable importance measures (VARIMP) for dogs of age 1–5 years for (**A**) loci Cfa6.6, Cfa6.7, and Cfa6.83 for separation distress; (**B**) loci Cfa6.6, Cfa6.7, and Cfa6.83 for attachment and attention-seeking; (**C**) all four loci for separation distress; and (**D**) all four loci for attachment and attention-seeking. VARIMP measures were computed using conditional random forests with 20,000 bootstrap samples.

To determine if specific allelic combinations across loci are more crucial in shaping the behavioral changes quantified by the C-BARQ©, we generated bootstrapped conditional random forest trees to assess nonparametric generalizations for R^2^ measures from the VARIMP scores. We found the highest positive variable importance for attachment/attention-seeking (VARIMP = 0.003) and separation-related problems (VARIMP = 0.04) at locus Cfa6.6 (Figure 1a,b). Locus Cfa6.7 had negative variable importance for attachment/attention-seeking (VIMP = −0.002) and positive variable importance for separation-related problems (VARIMP = 0.003), while locus Cfa6.83 had moderate positive variable importance for attachment/attention-seeking (VARIMP = 0.004) and separation-related problems (VARIMP = 0.001). When Cfa6.66 is included in the model, Cfa6.6 still has the highest variable importance (VARIMP = 0.012) for dogs of 1−5 years of age, but none of the loci have predictive power for attachment/attention-seeking (Figure 1c,d). 

### 3.3. Higher Mobile Element Copy Numbers at Cfa6.6 Co-Occur with Increased Hypersociability in Younger Dogs

**Figure 2:**
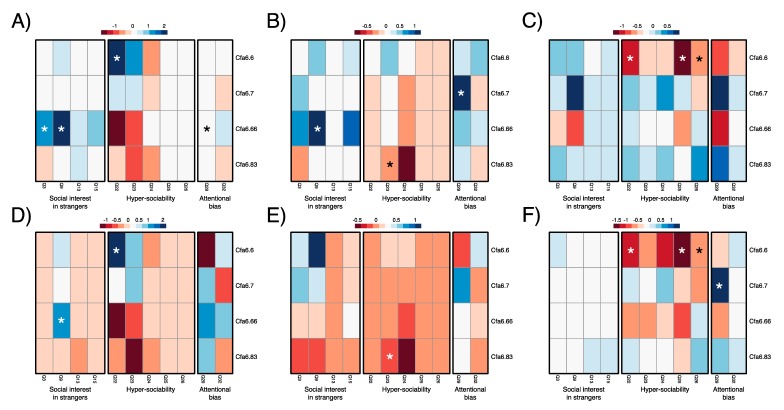
Heat map depicting beta values for associations between Cfa6.6, Cfa6.7, Cfa6.66, and Cfa6.83 MEI copy number and C-BARQ© scores assessing lower social interest in strangers (Q3, Q9, Q13, Q15), higher hypersociability (Q22, Q23, Q24, Q25, Q26), and lower attentional bias (Q29 and Q32) for (**A**) all dogs of 1–5 years of age (*n* = 117); (**B**) pet dogs of 1–5 years of age (*n* = 69); (**C**) pet dogs >5 years of age (*n* = 95); (**D**) all dogs owned for >1–5 years (*n* = 115); (**E**) pet dogs owned from 1–5 years (*n* = 65); and (**F**) pet dogs owned >5 years (*n* = 96). Significant beta values (*p* < 0.05) are marked with an asterisk (*). See Table S2 for more details on each insertion. C-BARQ©: Canine Behavioral Assessment Research Questionnaire; MEI: mobile element insertion.

Higher MEI copy number at Cfa6.6 is associated with decreased hypersociability for dogs of age >5 years (b = −1.167; *p* = 0.002) and those owned for >5 years (b = −1.215; *p* = 0.003) (Table 1). Contrary to this pattern, pets of age 1–5 years (b = 0.057; *p* = 0.242) and those owned from 1–5 years (b = 0.521; *p* = 0.206), have higher yet nonsignificant associations for MEI copy number at Cfa6.6 and increased hypersociability (Table 1). When we consider all dogs (assistance and pet) from ages 1–5, higher MEI copy number at Cfa6.6 is significantly associated with increased scores on hypersociability-related Q22 of the C-BARQ© (b = 2.168; *p* = 0.008) (Figure 2; Table S7). Higher MEI copy number at Cfa6.66 is significantly associated with higher scores on Q9, which is associated with increased aggression towards strangers (b = 1.120, *p* = 0.040) (Figure 2; Table S8). This association is also apparent for Q9 among assistance and pet dogs of 1–5 years of age (b = 1.859, *p* = 0.006), in addition to Q3 (b = 1.254, *p* = 0.029) (Figure 2; Table S7) and the overall behavioral summary of lower social interest in strangers (b = 0.238, *p* = 0.024) (Table 1). Higher MEI copy number at Cfa6.66 is associated with lower attentional bias on Q29 for pet dogs of age 1–5 years (b = 1.201; *p* = 0.025) and those owned from 1–5 years (b = 0.736, *p* = 0.029) (Figure 2; Table S9). This association with Q29 was also apparent for assistance and pet dogs of age 1–5 years (b = 0.156, *p* = 0.017) and those owned from 1–5 years (b = 1.184; *p* = 0.037) (Figure 2; Table S9), in addition to the overall behavioral summary of lower attention bias to stimuli (age 1–5 years: b = 1.239, *p* = 0.009; owned from 1–5 years: b = 0.656, *p* = 0.038) (Table 1).

### 3.4. Assistance Dogs Consistently Have More Mobile Element Insertions and Significant Heterozygosity Deficiency at Cfa6.6 Compared to Non-Assistance Dogs

**Figure 3:**
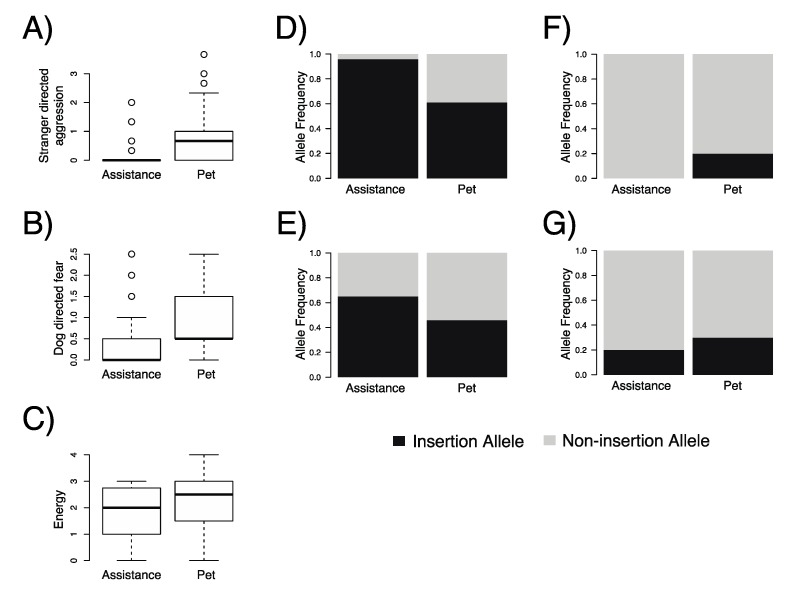
Mean and variation of C-BARQ© scores of behavioral types for assistance (*n* = 49) and pet (*n* = 69) dogs for (**A**) stranger-directed aggression; (**B**) dog-directed fear; and (**C**) energy. Bar graphs display the frequency of the inserted allele at each locus: (**D**) Cfa6.6; (**E**) Cfa6.7; (**F**) Cfa6.66; and (**G**) Cfa6.83.

German Shepherd assistance dogs of 1–5 years of age (*n* = 49) had significantly lower median scores on C-BARQ© questions negatively associated with social interest in strangers than pet dogs of the same age group (*n* = 69): stranger-directed aggression (median score: assistance = 0.0, pet = 0.7, *p* = 5.5 × 10^−7^; range: 0–4), dog-directed fear (median score: assistance = 0.0, pet = 0.5, *p* = 9.1 × 10^−4^; range: 0–4), and energy, which measures a dogs’ propensity to act in a playful, boisterous, and active manner (median score: assistance = 2.0, pet = 2.5, *p* = 0.03; range: 0–4) (Figure 3a,c). Assistance dogs had higher, yet nonsignificant, average C-BARQ© scores related to separation distress relative to pet dogs (average score: assistance = 0.8, pet = 0.3, *p* = 0.19; range: 0–4) (Table S10). 

Assistance dogs had lower observed heterozygosity (H_O_ = 0.08, *p* = 3.2 × 10^−4^) relative to pet dogs at loci Cfa6.6 (H_O_ = 0.48, *p* = 3.4 × 10^−4^), where the proportion of observed frequency of the insertion allele was 96% in assistance dogs, compared to 61% in pet dogs (Bonferroni-adjusted *p* = 2.3 × 10^−10^) (Figure 3d). Additionally, assistance dogs had significantly more MEIs at CFa6.7 (Fisher’s exact test; Bonferroni-adjusted *p* = 0.02) (Figure 3e). Further, German Shepherd assistance dogs had no alleles containing insertions at Cfa6.66, compared to the 20% MEI allele frequency in pet dogs (Fisher’s exact test; Bonferroni-adjusted *p* = 9.7 × 10^−7^) (Figure 3f). Retriever-clade assistance dogs had a lower heterozygosity (H_O_ = 0.378, *p* = 1.1 × 10^−8^) relative to pet dogs at locus Cfa6.6 (H_O_ = 0.471, *p* = 0.094). In comparison to pet Retriever-clade dogs (Labrador Retriever, *n* = 9; Golden Retriever, *n* = 48), assistance Retriever-clade individuals had a proportionally higher frequency of MEIs at Cfa6.6 (assistance = 75%, pet = 37%, Bonferroni-corrected *p* = 8.3 × 10^−12^) and Cfa6.66 (assistance = 18%, pet = 4%, Bonferroni-corrected *p* = 8.5 × 10^−4^), and lower frequency of MEIs at Cfa6.83 (assistance = 15%, pet = 54%, Bonferroni-corrected *p* = 1.1 × 10^−14^). For both sets of assistance dogs, the population were not in HWE and presented an excess of genotypes homozygous for the MEI at Cfa6.6 (Figure S2; Table 2).

### 3.5. Divergent-Breed Dogs Have Significantly More Mobile Element Insertions at Cfa6.6, Cfa6.7, and Cfa6.66

**Figure 4:**
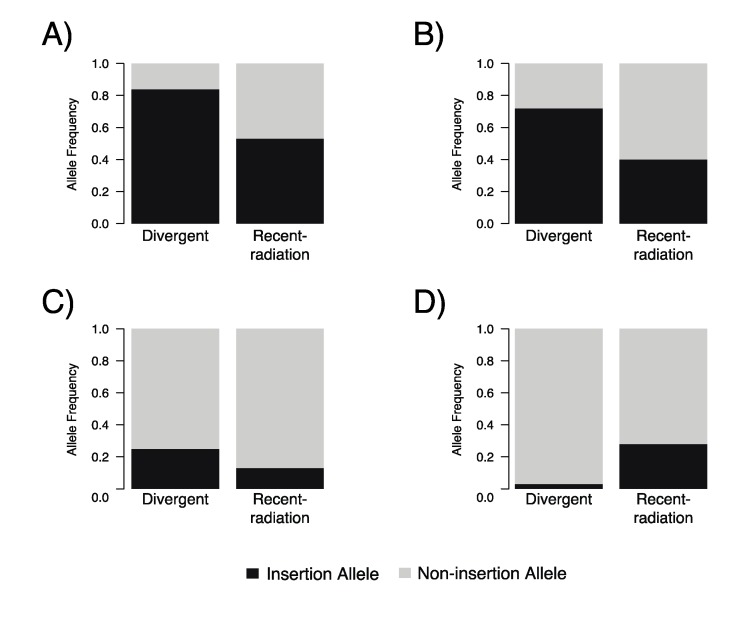
Frequency of the inserted allele within the divergent and recent-radiation breed groups, respectively, at each locus: (**A**) Cfa6.6; (**B**) Cfa6.7; (**C**) Cfa6.66; and (**D**) Cfa6.83.

Divergent dog breeds had higher frequency of inserted alleles at Cfa6.6 (Bonferroni-adjusted *p* = 3.6 × 10^−7^), Cfa6.7 (Bonferroni-adjusted *p* = 6.4 × 10^−7^), and Cfa6.66 (Bonferroni-adjusted *p* = 8.0 × 10^−7^) (Figure 4a,c); however, the opposite pattern was noted for locus Cfa6.83 (Bonferroni-adjusted *p* = 8.8 × 10^−8^) (Figure 4d). Additionally, we found a significant deficiency of heterozygosity for divergent dog breeds for loci Cfa6.6 (H_O_ = 0.276) and Cfa6.83 (H_O_ = 0.057), due to higher frequency of homozygous genotypes for the insertion at Cfa6.6 (*p* = 1.6 × 10^−3^) and homozygous genotypes for the no insertions at Cfa6.83 (*p* = 0.029).

## 4. Discussion

Our objective was to elucidate whether increased MEIs with hypersociability genes (*WBSCR17*, *GTF2I*, and *POM121*) on canine chromosome 6 (Cfa6) correspond to hypersocial behaviors in dogs as quantified by the C-BARQ©. We further investigated if the frequency of these MEIs differed between assistance and pet dogs to understand whether MEI screening of dogs could predict social behavior in adult behavior as reported by the C-BARQ©. Highly predictive genetic markers for social behaviors relevant to assistance dog training and placement may provide important benefits, including increased success rates, lower costs, and early behavioral interventions, allowing for better welfare and greater assistant dog availability. We discovered that locus Cfa6.6, a locus associated with *WBSCR17*, had the highest predictive power for C-BARQ© behaviors related to the hypersocial phenotype, which include higher levels of attention-seeking and separation-related distress among adult dogs of age 1–5 years. Increased frequencies of insertions at Cfa6.6 was associated with higher scores on the hypersociability-related C-BARQ© question Q22, which is consistent with previous work [22]. This question pertains to separation distress and assesses a dog’s restlessness, agitation, or pacing when about to be left alone by its owner. Due to low sample coverage per breed, we were unable to identify breed-specific variations in MEI frequency. This could have been informative in understanding breed-specific behaviors and potentially validating within-breed differences in MEI frequency and behavior. Nevertheless, our data is consistent with previously derived associations between proximity-based sociability and MEI frequency for dogs between 1–5 years of age. *WBSCR17* transcripts are known to be predominantly expressed in the cerebellum, hippocampus, thalamus, and cerebral cortex of the rat brain [40], with studies confirming its role in affecting cell morphology and cell membrane trafficking [41]. This is suggestive of a potential link between *WBSCR17* and behavior, although definitive proof of the functional link between *WBSCR17* and sociability is yet to be established. Furthermore, vonHoldt et al. and colleagues [24] discovered that Cfa6.6 is highly methylated, independent of MEI copy number. This is implicative of a more complex association between MEIs (at Cfa6.6) and *WBSCR17* expression than what was previously known.

One of the behavioral traits of successful assistance dogs include increased sociability towards people with disabilities [16]. We found that assistance dogs, of both German Shepherd and Retriever clades, showed reduced heterozygosity at Cfa6.6 relative to pet dogs due to the significant increase in the frequency of the insertion allele. Further, dogs homozygous for the insertion at Cfa6.6 also show increased human-directed hypersociability as quantified by separation distress and decreased human-opposed behavior such as stranger-directed aggression. Lormier and colleagues [42] found that canine fear and aggression are behaviors responsible for dismissal from assistance training programs. Currently, the costs of raising and training an assistance dog are as high as US$50,000 [18]. Genetic screening of dogs for insertions at *WBSCR17* may assist in identifying social predispositions of dogs early in development, which could increase successful training and placement of assistance dogs. Despite our analyses clearly showing higher frequency of MEIs in assistance dogs, comparative genetic and behavioral measures for successful versus unsuccessful assistance dogs could be useful for getting a clear measure of the predictive value for this proposed genetic testing. 

We hypothesize that hypersociability-associated insertions have changed in frequency over the history of dog domestication. Although we lack a direct survey of historical specimens, we combined dog breeds into groups that represented distinct genetic evolutionary histories. The group consisting of divergent dog breeds reflect a history that is distinct from breeds having a more recent augmentation and radiation, typical of European-derived breeds [34,35,36]. We suspect that hypersociability-associated insertions will be at a higher frequency within the divergent breed, reflecting the history of selection for human-directed friendliness, with a reduced frequency within the recent radiation that reflects a stronger selective pressure for breed function. Such breed functions may not reflect a selection of social behavior, but rather, task-focused behavior. Indeed, we found a higher frequency of MEIs at *WBSCR17* within divergent dog breeds. This finding is also consistent with the original discovery that *WBSCR17* contains genetic variation that differentiates domestic dogs from gray wolves [22,35], suggesting that behavioral traits were an important selection factor for dog domestication. Dog breeds that belong to the recent-radiation breed group represents rapid phenotypic divergence of breeds, after the implementation of the breed concept in early nineteenth-century Europe [34]. During the late 1800s, dog competitions evaluated dogs based on their physical appearances [34]. Prior to this, dog competitions focused on creating dogs that are good for hunting and chasing, rather than their physical appearance [39]. Therefore, the selection pressure for human-directed hypersociability in the recent radiation of breeds may not be as prominent as in antiquity. The complex domestication history of dogs suggests that insertions in *WBSCR17* would likely promote hypersocial behaviors, a trait that was most critical for assimilation into human societies.

While not a replacement for behavioral evaluations or interventions, genetic screening may provide an additional assessment tool to aid in the optimal placement, care, and training of animals based on underlying behavioral predispositions that may not otherwise be detectable early in life. Such a tool may be especially beneficial when evaluating working dog candidates, such as assistance dogs, for selective enrollment into resource-intensive training programs. From our analyses, we show that higher MEI copy number at *WBSCR17* is associated with C-BARQ© hypersociability behaviors and that these traits may be prominent in the tested populations of active and successful assistance dogs [6,20,21]. As a result, genetic screening for these hypersociability insertions may be a valuable tool in the early screening or breeding of assistance dogs.

## 5. Patents

This work is in support of the provisional patent #17-3362 (Title: Simple genomic test of canine genes associated with Williams–Beuren syndrome could predict social behavior in domesticated dogs), filed with Princeton University.

## Figures and Tables

**Table 1:** Per locus beta values as a function of MEI copy number and C-BARQ© score averages that quantify behaviors associated with three aspects of hypersociability as per vonHoldt et al. [22] Significance values are in parentheses (bolded values indicate *p* < 0.05).

Dataset Locus Name	Social Interest in Strangers *	Hypersociability	Attention Bias to Stimuli *
Assistance and pet dogs of 1–5 years of age (*n* = 117)			
Cfa6.6	0.071 (0.479)	0.609 (0.137)	0.093 (0.841)
Cfa6.7	0.029 (0.777)	0.257 (0.530)	0.001 (0.999)
Cfa6.66	**0.238 (0.024)**	−0.398 (0.345)	**1.239 (0.009)**
Cfa6.83	0.031 (0.762)	−0.257 (0.540)	0.085 (0.858)
Non-assistance dogs of 1–5 years of age (*n* = 69)			
Cfa6.6	0.328 (0.479)	0.057 (0.242)	0.495 (0.127)
Cfa6.7	0.260 (0.777)	−0.005 (0.924)	−0.097 (0.768)
Cfa6.66	0.771 (0.053)	−0.039 (0.442)	0.363 (0.319)
Cfa6.83	0.059 (0.882)	−0.082 (0.110)	−0.085 (0.816)
Non-assistance dogs >5 years of age (*n* = 95)			
Cfa6.6	−0.148 (0.708)	**−1.167 (0.002)**	−0.526 (0.217)
Cfa6.7	0.367 (0.353)	0.209 (0.592)	0.077 (0.856)
Cfa6.66	−0.392 (0.342)	−0.268 (0.500)	−0.273 (0.545)
Cfa6.83	0.186 (0.654)	0.137 (0.731)	0.006 (0.990)
Assistance and pet dogs owned for 1–5 years (*n* = 115)			
Cfa6.6	0.198 (0.562)	0.521 (0.206)	−0.210 (0.474)
Cfa6.7	0.064 (0.848)	0.349 (0.395)	−0.020 (0.944)
Cfa6.66	−0.592 (0.105)	−0.385 (0.359)	**0.656 (0.038)**
Cfa6.83	0.127 (0.723)	−0.248 (0.553)	0.152 (0.629)
Pet dogs owned for 1–5 years (*n* = 65)			
Cfa6.6	0.458 (0.208)	0.521 (0.206)	0.492 (0.131)
Cfa6.7	0.312 (0.391)	0.349 (0.395)	0.006 (0.984)
Cfa6.66	0.392 (0.335)	−0.385 (0.359)	0.092 (0.803)
Cfa6.83	−0.043 (0.914)	−0.248 (0.553)	−0.001 (0.997)
Non-assistance dogs owned for >5 years (*n* = 96)			
Cfa6.6	−0.025 (0.770)	**−1.215 (0.003)**	0.058 (0.804)
Cfa6.7	0.037 (0.668)	0.094 (0.821)	−0.071 (0.761)
Cfa6.66	−0.132 (0.137)	−0.571 (0.921)	−0.197 (0.415)
Cfa6.83	−0.060 (0.504)	0.042 (0.921)	0.192 (0.431)

* Note: Higher scores on respective C-BARQ© questions correspond to lower social interest in strangers and attention bias to stimuli (see Methods and Supplementary Table S5). Hence, positive beta values in those colunms correpond to lower and social interest in strangers and lower attention bias to stimuli with increased insertions (vice versa for negative beta values).

**Table 2:** Summary of per-locus significance testing from Hardy–Weinberg equilibrium exact tests for assistance and pet dogs. Genotypes are represented by the number of MEIs (homozygous with no insertions, 0; heterozygous for insertion, 1; homozygous for insertion, 2). Bolded values indicate *p* < 0.05. (Abbreviations: NP, test not performed)

Locus	Assistance Dogs (*n* = 196)				Pet Dogs (*n* = 126)				
	0	1	2	*p*-Value *	0	1	2	*p*-Value *	*p*-Value **
*Assistance (n* = *49) and pet dogs (n* = *69) of 1–5 years of age with C-BARQ© data*									
Cfa6.6	**0.04**	**0.00**	**0.96**	**3.3 × 10^−4^**	**0.22**	**0.34**	**0.44**	**0.021**	**2.3 × 10^−10^**
Cfa6.7	0.10	0.49	0.41	0.75	0.34	0.40	0.27	0.094	**0.02**
Cfa6.66	1.00	0.00	0.00	NP	**0.75**	**0.13**	**0.12**	**3.4 × 10^−5^**	**9.7 × 10^−7^**
Cfa6.83	0.63	0.33	0.04	1.00	**0.57**	**0.28**	**0.16**	**0.007**	0.53
*Assistance (n* = *147) and pet dogs (n* = *58) of the Retriever clade*									
Cfa6.6	**0.16**	**0.19**	**0.65**	**1.1 × 10^−8^**	0.19	0.36	0.45	0.094	**2.1 × 10^−12^**
Cfa6.7	0.48	0.41	0.11	0.566	0.03	0.47	0.03	0.309	1
Cfa6.66	0.69	0.27	0.04	0.400	0.93	0.05	0.02	0.085	**8.5 × 10^−4^**
Cfa6.83	**0.79**	**0.13**	**0.08**	**5.4 × 10^−7^**	**0.40**	**0.29**	**0.40**	**0.002**	**1.1 × 10^−14^**

* *p*-value derived from Hardy–Weinberg equilibrium exact tests, assessing within-group allele frequencies against the HWE null hypothesis; ** *p*-value derived from Fisher’s exact test, assessing differences in allele frequencies between assistance and pet dogs.

## Supplementary Materials

File S1: The following are available online at https://www.mdpi.com/2073-4425/10/6/439/s1, Table S1: Number of dogs genotyped and with C-BARQ© behavioral data, Table S2: Primer sequence and amplicon information for four retro-transposon mobile element insertions located on canine chromosome 6 in Canfam3.1, Table S3: Thermocycling reagents and concentrations for locus amplification using either Protocol 1 with cycling conditions: 95 °C for 10 min; 30 cycles of 95 °C for 30 s, 60 °C for 30 s, 72 °C for 45 s; 72 °C for 10 min; 4 °C hold or Protocol 2. Cycling conditions with cycling conditions: 95 °C for 5 min; 46 cycles of 94 °C for 1 min, 60 °C for 1 min, 72 °C for 1.5 min; 72 °C for 5 min; 4 °C hold, Table S4: Calculation of C-BARQ© behavior axes as per averaging scores for the below identified questions, Table S5: Questions on the C-BARQ© that were tested for overlap in behaviors found to be associated with canine hypersociability by vonHoldt et al., Table S6: Classification of breed groups, Table S7, Beta values for locus-specific MEI copy number and C-BARQ© score averages informative for decreasing social interest in strangers, and for relevant questions tagging this behavioral summary, Table S8: Beta values for locus-specific MEI copy number and C-BARQ© score averages informative for increasing hypersociability, and for relevant questions tagging this behavioral summary, Table S9: Beta values for locus-specific MEI copy number and C-BARQ© score averages informative for decreasing attention bias to stimuli, and for relevant questions tagging this behavioral summary, Table S10: Differences in scores between assistance dogs and pet dogs on the C-BARQ© with respect to the defined behavioral axes. Figure S1: Correlation between age and C-BARQ©: (A) attachment/attention seeking; (B) separation-related problems; and (C) stranger-directed aggression, Figure S2: Frequency of the inserted allele in Retriever-clade assistance (*n* = 147) and pet dogs (*n* = 58) at each locus: (A) Cfa6.6; (B) Cfa6.7; (C) Cfa6.66; and (D) Cfa6.83, Figure S3. Pairwise correlations between the 14 behavioral axes of the C-BARQ©. File S2: Raw Data Containing genotypic data for 837 dogs and paired C-BARQ© scores 228 dogs, File S3: Short version of the C-BARQ© questionnaire used in this study

## References

[1] Cadieu E., Neff M.W., Quignon P., Walsh K., Chase K., Parker H.G., vonHoldt H.G., Rhue A., Boyko A., Byers A. (2009). Coat Variation in the Domestic Dog Is Governed by Variants in Three Genes. Science.

[2] Parker H.G., VonHoldt B.M., Quignon P., Margulies E.H., Shao S., Mosher D.S., Spady T.C., Elkahloun A., Cargill M., Jones P.G. (2009). An expressed fgf4 retrogene is associated with breed-defining chondrodysplasia in domestic dogs. Science.

[3] Boyko A.R., Quignon P., Li L., Schoenebeck J.J., Degenhardt J.D., Lohmueller K.E., Zhao K., Brisbin A., Parker H.G., vonHoldt B.M. (2010). A Simple Genetic Architecture Underlies Morphological Variation in Dogs. PloS Biol..

[4] Menchetti L., Righi C., Guelfi G., Enas C., Moscati L., Mancini S., Diverio S. (2019). Multi-Operator Qualitative Behavioural Assessment for dogs entering the shelter. Appl. Anim. Behav. Sci..

[5] Bremhorst A., Mongillo P., Howell T., Marinelli L. (2018). Spotlight on Assistance Dogs-Legislation, Welfare and Research. Animals.

[6] Bray E.E., Levy K.M., Kennedy B.S., Duffy D.L., Serpell J.A., Maclean E.L. (2019). Predictive Models of Assistance Dog Training Outcomes Using the Canine Behavioral Assessment and Research Questionnaire and a Standardized Temperament Evaluation. Front. Vet. Sci..

[7] Chopik J.W., Weaver J.R. (2019). Old dog, new tricks: Age differences in dog personality traits, associations with human personality traits, and links to important outcomes. J. Res. Pers..

[8] Serpell J. (1995). The Domestic Dog: Its Evolution, Behaviour and Interactions with People.

[9] Jones A.C., Gosling S.D. (2005). Temperament and personality in dogs (*Canis familiaris*): A review and evaluation of past research. Appl. Anim. Behav. Sci..

[10] Miklόsi A. (2015). Dog Behaviour, Evolution, and Cognition.

[11] Goddard M.E., Beilharz R.G. (1984). The relationship of fearfulness to, and the effects of, sex, age and experience on exploration and activity in dogs. Appl. Anim. Behav. Sci..

[12] Murphy J.A. (1998). Describing categories of temperament in potential guide dogs for the blind. Appl. Anim. Behav. Sci..

[13] Maejima M., Inoue-Murayama M., Tonosaki K., Matsuura N., Kato S., Saito Y., Weiss A., Murayama Y., Ito S. (2007). Traits and genotypes may predict the successful training of drug detection dogs. Appl. Anim. Behav. Sci..

[14] Hsu Y., Serpell J.A. (2003). Development and validation of a questionnaire for measuring behavior and temperament traits in pet dogs. JAVMA.

[15] Allen K., Blascovich J. (1996). The value of service dogs for people with severe ambulatory disabilities. A randomized controlled trial. JAMA.

[16] Audrestch H.M., Whelan C.T., Grice D., Asher L., England G.C., Freeman S.L. (2015). Recognizing the value of assistance dogs in society. Disabil. Health J..

[17] Sachs-Ericsson N., Hansen N.K., Fitzgerald S. (2002). Benefits of assistance dogs: A review. Rehabil. Psychol..

[18] Byrne C., Zuerndorfer J., Freil L., Han X., Sirolly A., Gilliland S., Starner T., Jackson M. (2018). Predicting the Suitability of Service Animals Using Instrumented Dog Toys. IMWUT.

[19] Ostrander E.A.

[20] Arata S., Momozawa Y., Takeuchi Y., Mori Y. (2010). Important behavioral traits for predicting guide dog qualification. J. Vet. Med. Sci..

[21] Duffy D.L., Serpell J.A. (2008). Behavioral assessment of guide and service dogs. J. Vet. Med. Sci..

[22] VonHoldt B.M., Shuldiner E., Janowitz I.K., Kartzinel R.Y., Hogan A., Brubaker B., Wanser S., Stahler D., Wynne C.D., Ostrander E.A. (2017). Structural variants in genes associated with human Williams-Beuren Syndrome underlie stereotypical hyper-sociability in domestic dogs. Sci. Adv..

[23] Shubert C. (2009). The genomic basis of the Williams—Beuren syndrome. Cell Mol. Life Sci..

[24] vonHoldt B.M., Ji S.S., Aardema M.L., Stahler D.R., Udell M.A.R., Sinsheimer J.S. (2018). Activity of genes with functions in human William-Beuren Syndrome is impacted by mobile element insertions in the gray wolf genome. Genome Biol. Evol..

[25] Kajikawa M., Okada N. (2002). LINEs mobilize SINEs in the eel through a shared 3′ sequence. Cell.

[26] Hothorn T., Buehlmann P., Dudoit S., Molinaro A., Laan M.D.V. (2006). Survival Ensembles. Biostatistics.

[27] Strobl C., Boulesteix A.L., Zeileis A., Hothorn T. (2007). Bias in random forest variable importance measures: Illustrations, sources and a solution. BMC Bioinform..

[28] Strobl C., Boulesteix A.L., Kneib T., Augustin T., Zeileis A. (2008). Conditional Variable Importance for Random Forests. BMC Bioinform..

[29] Breiman L. (2001). Random Forests. Mach. Learn..

[30] Hoerl A.E., Kennard R.W. (1970). Biased Estimation for Nonorthogonal Problems. Technometrics.

[31] Cule E., Iorio M.D. (2013). Ridge regression in prediction problems: Automatic choice of the ridge parameter. Genet. Epidemiol..

[32] R: A language and environment for statistical computing. 2017 R Foundation for Statistical Computing, Vienna, Austria. https://www.R-project.org/.

[33] Warnes G., Gorjanc G., Leisch F., Man M. (2019). Population Genetics. R package version 1.3.8.1.2. https://cran.r-project.org/web/packages/genetics/index.html.

[34] Parker H.G., Kim L.V., Sutter N.B., Carlson S., Lorentzen T.D., Malek T.B., Johnson G.S., DeFrance H.B., Ostrander E.A., Kruglyak L. (2004). Genetic structure of the purebred domestic dog. Science.

[35] VonHoldt B.M., Pollinger J.P., Lohmueller K.E., Han E., Parker H.G., Quignon P., Degenhardt J.D., Boyko A.R., Earl D.A., Auton A. (2010). Genome-wide SNP and haplotype analyses reveal a rich history underlying dog domestication. Nature.

[36] Parker H.G., Dreger D.L., Rimbault M., Davis B.W., Mullen A.B., Carpintero-Ramirez G., Ostrander E.A. (2017). Genomic Analyses Reveal the Influence of Geographic Origin, Migration, and Hybridization on Modern Dog Breed Development. Cell Rep..

[37] Wilcox B., Walkowicz C. (1995). The Atlas of Dog Breeds of the World.

[38] Bruce F. (2000). The New Encyclopedia of the Dog.

[39] American Kennel Club (2006). The Complete Dog Book.

[40] Nakamura N., Toba S., Hirai M., Morishita S., Mikami T., Konishi M., Itoh N., Kurosaka A. (2005). Cloning and expression of a brain-specific putative UDP-GalNAc:polypeptide N-acetylgalactosaminyltransferase gene. Biol. Pharm. Bull..

[41] Nakayama Y., Nakamura N., Oki S., Wakabayashi M., Ishihana Y., Miyake A., Itoh N., Kurosaka A. (2012). A putative polypeptide N-acetylgalactosaminyltransferase/Williams-Beuren syndrome chromosome region 17 (*WBSCR17*) regulates lamellipodium formation and macropinocytosis. J. Biol. Chem..

[42] Lorimer G.C., Harvey N.D., England G.C.W., Aher L. (2016). Using the incidence and impact of behavioural conditions in guide dogs to investigate patterns in undesirable behaviour in dogs. Sci. Rep. UK.

