# Supplementary material for: Homozygosity for Mobile Element Insertions Associated with WBSCR17 Could Predict Success in Assistance Dog Training Programs
Source: Genes (Basel). 2019 Jun 9;10(6):439. doi: 10.3390/genes10060439 (PMC6627829; doi:10.3390/genes10060439)
Supplement: Supplementary file 1 [file genes-10-00439-s001.zip › genes-504842 supplementary -final/File S1.docx]

**Supplementary Table S1.** Number of dogs genotyped and with C-BARQ© behavioral data. (Abbreviations: f, frequency; MEI, mobile element insertion; n, sample size; ND, no data; Unk, unknown).

|  | ***f*(MEI)** | **Samples with C-BARQ data** | | | | | | | | |
| --- | --- | --- | --- | --- | --- | --- | --- | --- | --- | --- |
| **Group** | **Total n (C-BARQ data, n)** | **Cfa6.6** | **Cfa6.7** | **Cfa6.66** | **Cfa6.83** | **1-5 years old (n)** | **>5 years old (n)** | **Owned for 1-5 years (n)** | **Owned for >5 years (n)** | **Unk (Age)** |
| All dogs | 837 (228) | 1104 | 759 | 245 | 371 | 117 | 95 | 65 | 98 | 17 |
| Assistance dogs | 196 (49) | 314 | 156 | 52 | 63 | 48 | 0 | 48 | 0 | - |
| German shepherd | 49 (49) | 94 | 64 | 0 | 20 | 49 | 0 | 49 | 0 | - |
| Golden retrievers | 29 (0) | 10 | 2 | 7 | 37 | 0 | 0 | 0 | 0 | - |
| Labrador retrievers | 118 (0) | 210 | 90 | 45 | 6 | 0 | 0 | 0 | 0 | - |
| Non-assistance dogs | 641 (181) | 790 | 603 | 193 | 308 | 69 | 95 | 65 | 98 | 17 |
| Breed groups* |  |  |  |  |  |  |  |  |  |  |
| Antiquity | 52 (2) | 87 | 75 | 26 | 3 | - | - | - | - | - |
| Victorian era | 324 (91) | 345 | 269 | 69 | 162 | - | - | - | - | - |

* Groups were based on those reported in vonHoldt et al. (2010) and Parker et al. (2017). Assistance dogs were excluded from the breed group analyses. Only genotype analysis was conducted for breed groups, due to lack of C-BARQ data for ‘antiquity’ breed dogs. **The following breeds were not classified as either Antiquity or Victorian era in vonHoldt et al. and Parker et al. 2004 were excluded from the analyses looking at breed group differences: 1) Australian Labradoodle, 2) Boykin Spaniel, 3) Boz Guregh, 4) Catahoula Cur, 5) Cockapoo, 6) Cocker Spaniel, 7) Connemara Terrier, 8) English Pointer, 9) English Shepherd, 10) Golden Doodle, 11) Irish Jack Russel, 12) Miniature Dachshund, 13) Pariah, 14) Pyrenees Shepherd, and 15) Mix Breeds.

**Supplementary Table S2.** Primer sequence and amplicon information for four retro-transposon mobile element insertions located on canine chromosome 6 in Canfam3.1. (Abbreviations: bp, base pair).

| **Locus name** | Start position | Associated gene | Primer sequence (5’ -> 3’) | Amplicon size (bp): | |
| --- | --- | --- | --- | --- | --- |
|  |  |  |  | Insertion | Wildtype |
| Cfa6.6 | 2,521,650 | *WBSCR17* | Forward: CCCCTTCAGCCAGCATATAA  Reverse: TTCTCTGGGCTGTCTGGACT | 555 | 357 |
| Cfa6.7 | 2,546,359 | *WBSCR17* | Forward: TGGAGCCATGATTAGGAAGG  Reverse: TAAGGAAGGACCCCATTTCC | 504 | 269 |
| Cfa6.66 | 5,753,706 | *GTF2I* | Forward: TGCTGCTTCATGTTCTGTGA  Reverse: TGGTGCATTAGCTTTGGTTG | 505 | 215 |
| Cfa6.83 | 6,914,106 | *POM121* | Forward: AACCACAGGAACAAAACCTCA  Reverse: CCTCCTGTTGGACATTTGGA | 400 | 184 |

**Supplementary Table S3.** Thermocycling reagents and concentrations for locus amplification using either Protocol 1 with cycling conditions: 95 °C for 10min; 30 cycles of 95 °C for 30s, 60 °C for 30s, 72 °C for 45s; 72 °C for 10min; 4 °C hold or Protocol 2. Cycling conditions with cycling conditions: 95 °C for 5min; 46 cycles of 94 °C for 1min, 60 °C for 1min, 72 °C for 1.5min; 72 °C for 5min; 4 °C hold. The desired PCR products are between 200bp-500bp. Protocol 1 amplifies loci with higher specificity, while Protocol 2 is used when DNA volume is limited.

| **Protocol** | **Amount per reaction** |
| --- | --- |
| Protocol 1 | 0.15µl BSA (10mg/mL) |
|  | 0.50µl DMSO |
|  | 0.30µl Forward primer (10uM) |
|  | 0.30µl Reverse primer (10uM) |
|  | 2.25µl molecular grade water (nuclease-free) |
|  | 7.50µl MyTaq HS Red Mix (New England Biolabs) |
|  | 4µl DNA (1-3ng/ul) |
| Protocol 2 | 0.15µl BSA (10mg/mL) |
|  | 0.50µl DMSO |
|  | 0.30µl Forward primer (10uM) |
|  | 0.30µl Reverse primer (10uM) |
|  | 4.25µl molecular grade water (nuclease-free) |
|  | 7.50µl MyTaq HS Red Mix (New England Biolabs) |
|  | 2µl DNA (1-3ng/ul) |

**Supplementary Table S4.** Calculation of C-BARQ© behavior axes as per averaging scores for the below identified questions.

| **Behavioral Summary** | **Questions (Q)** |
| --- | --- |
| Excitability | Q1, Q2 |
| Stranger directed aggression | Q3, Q6, Q9 |
| Dog directed aggression | Q4, Q5, Q7 |
| Owner directed aggression | Q8, Q10 |
| Familiar dog aggression | Q11, Q12 |
| Stranger directed fear | Q13, Q15 |
| Nonsocial fear | Q14, Q16, Q18 |
| Dog-directed fear | Q17, Q19 |
| Touch sensitivity | Q20, Q21 |
| Separation distress | Q22, Q23, Q24 |
| Attachment/attention seeking | Q25, Q26 |
| Training difficulty | Q27, Q28, Q29 |
| Chasing | Q30, Q31 |
| Energy | Q39, Q40 |

**Supplementary Table S5.** Questions on the C-BARQ© that were tested for overlap in behaviors found to be associated with canine hyper-sociability by vonHoldt et al. (Abbreviations: Q, question).

| **C-BARQ© questions** | **C-BARQ© behavior** | **Behavioral assay** |
| --- | --- | --- |
| Q29, Q32 | Tendency of dog to become distracted by interesting [sounds/smells/birds] stimuli | Attentional bias: the amount of time a dog spends looking at a human, as opposed to another non-social stimulus such as a puzzle |
| Q22, Q23, Q24 | Tendency of dog to become anxious when left along by their owners | Hypersociability: the amount of time the dog spends in the proximity of a person |
| Q25, Q26 | Tendency of dog to follow owners around the house and sit closely to household members | Hypersociability: the amount of time the dog spends in the proximity of a person |
| Q3, Q9, Q13, Q15 | Tendency of dog to become aggressive or fearful when approached by strangers | Social interest in strangers: the amount of time a dog spends in the proximity of an unfamiliar person and assesses the engagement of a dog with strangers |

**Supplementary Table S6.** Classification of breed groups.

| Breed group | Sample size | Breeds |
| --- | --- | --- |
| Divergent | 52 | Afghan hound, Akita, Basenji, Chow-chow, Eurasier, Finnish Spitz, Greenland sled dog, Ibizan hound, Kuvasz, Malamute, Saluki, Samoyed, Shar-Pei, Shiba Inu, Siberian Husky, Tibetan Mastiff, Xigou |
| Recent radiation | 324 | Airedale Terrier, American Cocker Spaniel, American Eskimo, American Hairless Terrier, American Staffordshire Terrier, Anatolian Shepherd, Australian Shepherd, Australian Silkie Terrier, Australian Terrier, Australian Cattle dog, Azawakh, Basset Hound, Beagle, Bearded Collie, Bedlington Terrier, Belgian Malinois, Belgian Tervuren, Berger Picard, Berenese Mountain dog, Bichon Frise, Black Russian Terrier, Bloodhound, Boerboel, Border Collie, Border Terrier, Borzoi, Boston Terrier, Bouvier des Flandres, Boxer, Briard, Brittany Spaniel, Brussels Griffon, Bull Terrier, Bulldog, Bullmastiff, Cairn Terrier, Cane Corso, Cardigan Welsh Corgi, Cavalier King Charles Spaniel, Chihuahua, Chinese Crested, Chinook, Cirneco del Etna, Collie, Coto du Tulear, Curly Coated Retriever, Dachshund, Dalmatian, Doberman, Dogue de Boreaux, English Cocker Spaniel, English Setter, English Springer Spaniel, Field Spaniel, Flat-Coated Retriever, Fox Hound, French Bulldog, German Shepherd, German Short-haired Pointer, German Wirehaired Pointer, Glen of Imaal Terrier, Golden Retriever, Gordon Setter, Great Dane, Great Pyrenees, Greater Swiss Mountain dog, Greyhound, Great Schnauzer, Havanese, Icelandic Sheepdog, Irish Setter, Irish Terrier, Irish Water Spaniel, Irish Wolfhound, Italian Greyhound, Jack Russel, Japanese Chin, Keeshond, Kelpie, Kerry Blue Terrier, Komonder, Labrador Retriever, Large Musterlander, Leonberger, Levriero Meridinale, Lhasa Apso, Maltese, Mastiff, Mastino Abruzzese, Miniature Bull Terrier, Mini Poodle, Miniature Pinscher, Miniature Schnauzer, Miniature Xoloitzcuintli, Neapolitan Mastiff, Newfoundland, Norwegian Elkhound, Norfolk Terrier, Norwich Terrier, Nova Scotia Duck Tolling Retriever, Old English Sheepdog, Otterhound, Papillion, Pharaoh Hound, Parsons Russel Terrier, Pekingese, Pembroke Welsh Corgi, Peruvian Hairless, Petit Basset Griffon Vendeen, Pomeranian, Portuguese Water dog, Pug, Puli, Pumi, Rat Terrier, Redbone Coonhound, Rhodesian Ridgeback, Rottweiler, Scottish Deerhound, Scottish Terrier, Shetland Sheepdog, Shih Tzu, Sloughi, Soft Coated Wheaton Terrier, Spinone Italiano, Saint Bernard, Stafford Bull Terrier, Standard Poodle, Standard Schnauzer, Swedish Valhund, Tibetan Spaniel, Tibetan Terrier, Toy Fox Terrier, Toy Manchester Terrier, Toy Poodle, Vizsla, Weimaraner, West Highland White Terrier, Whippet, Wire Fox Terrier, Wirehaired Pointing Griffon, Xoloitzcuintli, Yorkshire Terrier |

| Dataset  Locus Name | Hyper- sociability | Q22 | Q23 | Q24 | Q25 | Q26 |
| --- | --- | --- | --- | --- | --- | --- |
| All dogs between 1-5 years of age (n=117) | | | | | | |
| Cfa6.6 | 0.609 (0.137 ) | **2.168 (0.008)** | 1.080 (0.089) | -0.552 (0.245) | 0.001 (0.879) | 0.002 (0.862) |
| Cfa6.7 | 0.257 (0.530 ) | 0.411 (0.669) | 0.650 (0.308) | -0.165 (0.728) | -0.001 (0.864) | -0.012 (0.474) |
| Cfa6.66 | -0.398 (0.345) | **-1.934 (0.013)** | -0.846 (0.200) | 0.252 (0.599) | 0.001 (0.808) | 0.002 (0.903) |
| Cfa6.83 | -0.257 (0.540) | -0.291 (0.709) | -1.085 (0.100) | -0.653 (0.174) | -0.001 (0.819) | -0.008 (0.629) |
| Pet Dogs between 1-5 years of age (n=69) | | | | | | |
| Cfa6.6 | 0.057 (0.242) | 0.074 (0.236) | 0.603 (0.057) | 0.122 (0.783) | 0.029 (0.552) | 0.005 (0.844) |
| Cfa6.7 | -0.005 (0.924) | 0.011 (0.855) | 0.187 (0.558) | -0.305 (0.495) | -0.001 (0.981) | -0.022 (0.394) |
| Cfa6.66 | -0.039 (0.442) | -0.094 (0.146) | 0.066 (0.158) | -0.294 (0.538) | -0.001 (0.984) | 0.011 (0.668) |
| Cfa6.83 | -0.082 (0.110) | -0.027 (0.678) | **-0.301 (0.048)** | -0.937 (0.050) | -0.031 (0.527) | -0.017 (0.518) |
| Pet dogs >5 years of age (n=95) | | | | | | |
| Cfa6.6 | **-1.167 (0.002)** | **-1.140 (0.009)** | -0.526 (0.217) | **-0.526 (0.010)** | **-1.350 (0.025)** | -0.611 (0.190) |
| Cfa6.7 | 0.209 (0.592) | 0.178 (0.684) | 0.077 (0.856) | 0.304 (0.140) | -0.117 (0.846) | -0.383(0.412) |
| Cfa6.66 | -0.268 (0.500) | -0.055 (0.903) | -0.273 (0.545) | -0.120 (0.581) | -0.719 (0.250) | 0.080 (0.868) |
| Cfa6.83 | 0.137 (0.731) | 0.211 (0.646) | 0.006 (0.990) | 0.139 (0.525) | -0.245 (0.696) | 0.429 (0.377) |
| All dogs owned from 1-5 years (n=115) | | | | | | |
| Cfa6.6 | 0.521 (0.206) | **1.789 (0.024)** | 0.870 (0.152) | -0.337 (0.300) | 0.001 (0.744) | 0.000 (0.961) |
| Cfa6.7 | 0.349 (0.395) | 0.286 (0.720) | 0.660 (0.274) | 0.077 (0.812) | -0.000 (0.991) | -0.004 (0.562) |
| Cfa6.66 | -0.385 (0.359) | -1.333 (0.082) | -0.606 (0.336) | 0.056 (0.872) | 0.000 (0.827) | -0.005 (0.511) |
| Cfa6.83 | -0.248 (0.553) | -0.313 (0.634) | -1.174 (0.061) | -0.346 (0.319) | -0.000 (0.995) | -0.002 (0.751) |
| Pet dogs owned from 1-5 years (n=65) | | | | | | |
| Cfa6.6 | 0.001 (0.345) | 0.000 (0.548) | 0.095 (0.176) | 0.083 (0.769) | 0.034 (0.451) | 0.015 (0.807) |
| Cfa6.7 | -0.000 (0.973) | -0.000 (0.771) | 0.038 (0.587) | 0.049 (0.862) | 0.005 (0.914) | -0.038 (0.530) |
| Cfa6.66 | -0.001 (0.357) | -0.000 (0.342) | -0.078 (0.290) | -0.241 (0.452) | -0.002 (0.962) | -0.026 (0.675) |
| Cfa6.83 | -0.001 (0.138) | -0.000 (0.713) | **-0.154 (0.034)** | -0.563 (0.092) | -0.022 (0.633) | -0.036 (0.564) |
| Pet dogs owned >5 years (n=96) | | | | | | |
| Cfa6.6 | **-1.215 (0.003)** | **-1.042 (0.011)** | -0.575 (0.275) | **-1.062 (0.005)** | **-1.568 (0.018)** | -0.656 (0.152) |
| Cfa6.7 | 0.094 (0.821) | 0.045 (0.912) | -0.104 (0.843) | 0.369 (0.327) | -0.469 (0.478) | -0.625 (0.173) |
| Cfa6.66 | -0.571 (0.921) | -0.539 (0.206) | -0.661 (0.228) | -0.406 (0.297) | -0.859 (0.205) | 0.158 (0.173) |
| Cfa6.83 | 0.042 (0.921) | 0.237 (0.581) | -0.034 (0.950) | -0.073 (0.853) | -0.330 (0.628) | 0.378 (0.427) |

**Supplementary Table S7.** Beta values for locus-specific MEI copy number and C-BARQ© score averages informative for hyper-sociability and for relevant question tagging this behavioral summary. *P*-values are in parentheses; bolded values indicate *p*<0.05. Beta values were obtained using linear ridge regression with age and sex as covariates.

**Supplementary Table S8.** Beta values for locus-specific MEI copy number and C-BARQ© score averages informative for social interest in strangers and for relevant question tagging this behavioral summary. *P*-values are in parentheses; bolded values indicate *p*<0.05. Beta values were obtained using linear ridge regression with age and sex as covariates.

| Dataset  Locus Name | Social interest in strangers | Q3 | Q9 | Q13 | Q15 |
| --- | --- | --- | --- | --- | --- |
| All dogs between 1-5 years of age (n=117) | | | | | |
| Cfa6.6 | 0.071 (0.479) | 0.161 (0.772) | 0.431 (0.533) | 0.301 (0.526) | 0.218 (0.533) |
| Cfa6.7 | 0.029 (0.777) | -0.057 (0.970) | 0.019 (0.978) | 0.121 (0.799) | 0.066 (0.850) |
| Cfa6.66 | **0.238 (0.024)** | **1.254 (0.029)** | **1.859 (0.006)** | 0.375 (0.460) | 0.745 (0.051) |
| Cfa6.83 | 0.031 (0.762)(0.762) | -0.102 (0.859) | 0.114 (0.865) | 0.364 (0.469) | 0.194 (0.606) |
| Pet Dogs between 1-5 years of age (n=69) | | | | | |
| Cfa6.6 | 0.328 (0.479) | 0.168 (0.693) | 0.534 (0.277) | 0.149 (0.532) | 0.390 (0.449) |
| Cfa6.7 | 0.260 (0.777) | 0.452 (0.289) | 0.222 (0.654) | 0.124 (0.604) | 0.206 (0.691) |
| Cfa6.66 | 0.771 (0.053) | 0.804 (0.086) | **1.120 (0.040)** | 0.120 (0.454) | 0.907 (0.111) |
| Cfa6.83 | 0.059 (0.882) | -0.230 (0.625) | 0.130 (0.812) | 0.055 (0.838) | 0.220 (0.700) |
| Pet dogs >5 years of age (n=95) | | | | | |
| Cfa6.6 | -0.148 (0.708) | 0.173 (0.704) | 0.095 (0.874) | -0.208 (0.295) | -0.000 (0.743) |
| Cfa6.7 | 0.367 (0.353) | 0.059 (0.898) | 0.767 (0.198) | 0.064 (0.749) | 0.000 (0.521) |
| Cfa6.66 | -0.392 (0.342) | -0.340 (0.475) | -0.885 (0.151) | -0.041 (0.841) | -0.001 (0.417) |
| Cfa6.83 | 0.186 (0.654) | 0.292 (0.539) | 0.046 (0.940) | 0.057 (0.784) | -0.000 (0.538) |
| All dogs owned from 1-5 years (n=115) | | | | | |
| Cfa6.6 | 0.198 (0.562) | 0.000 (0.669) | 0.580 (0.366) | 0.017 (0.300) | 0.001 (0.744) |
| Cfa6.7 | 0.064 (0.848) | 0.000 (0.888) | 0.292 (0.647) | 0.077 (0.812) | -0.000 (0.991) |
| Cfa6.66 | -0.592 (0.105) | 0.000 (0.151) | 0.948 (0.148) | 0.056 (0.872) | 0.000 (0.827) |
| Cfa6.83 | 0.127 (0.723) | 0.000 (0.962) | -0.142 (0.828) | -0.346 (0.319) | -0.000 (0.995) |
| Pet dogs owned from 1-5 years (n=65) | | | | | |
| Cfa6.6 | 0.458 (0.208) | 0.531 (0.168) | 1.187 (0.050) | -0.000 (0.777) | 0.129 (0.506) |
| Cfa6.7 | 0.312 (0.391) | 0.562 (0.141) | 0.497 (0.412) | -0.000 (0.812) | 0.086 (0.658) |
| Cfa6.66 | 0.392 (0.335) | 0.219 (0.617) | 0.218 (0.740) | 0.001 (0.872) | 0.253 (0.236) |
| Cfa6.83 | -0.043 (0.914) | -0.219 (0.615) | -0.236 (0.720) | 0.000 (0.319) | 0.059 (0.781) |
| Pet dogs owned >5 years (n=96) | | | | | |
| Cfa6.6 | -0.025 (0.770) | 0.031 (0.734) | -0.000 (0.985) | -0.095 (0.438) | -0.036 (0.707) |
| Cfa6.7 | 0.037 (0.668) | -0.003 (0.970) | 0.009 (0.371) | 0.021 (0.866) | 0.026 (0.787) |
| Cfa6.66 | -0.132 (0.137) | -0.082 (0.382) | -0.013 (0.185) | -0.123 (0.329) | -0.141 (0.141) |
| Cfa6.83 | -0.060 (0.504) | -0.029 (0.832) | 0.005 (0.597) | 0.064 (0.609) | 0.080 (0.409) |

**Supplementary Table S9.** Beta values for locus-specific MEI copy number and C-BARQ© score averages informative for attention bias to stimuli and for relevant question tagging this behavioral summary. *P*-values are in parentheses; bolded values indicate *p*<0.05. Beta values were obtained using linear ridge regression, using age and sex as covariates.

| **Dataset**  **Locus Name** | **Attention bias to stimuli** | **Q29** | **Q32** |
| --- | --- | --- | --- |
| All dogs between 1-5 years of age (n=117) | | | |
| Cfa6.6 | 0.093 (0.841) | -0.012 (0.772) | 0.197 (0.533) |
| Cfa6.7 | 0.001 (0.999) | 0.080 (0.209) | -0.397 (0.589) |
| Cfa6.66 | **1.239 (0.009)** | **0.156 (0.017)** | 0.589 (0.086) |
| Cfa6.83 | 0.085 (0.858) | 0.062 (0.344) | -0.224 (0.508) |
| Pet Dogs between 1-5 years of age (n=69) | | | |
| Cfa6.6 | 0.649 (0.104) | 0.405 (0.450) | 0.495 (0.127) |
| Cfa6.7 | 0.390 (0.332) | **1.201 (0.025)** | -0.097 (0.768) |
| Cfa6.66 | 0.490 (0.261) | 0.466 (0.416) | 0.363 (0.319) |
| Cfa6.83 | 0.059 (0.897) | 0.256 (0.654) | -0.085 (0.816) |
| Pet dogs >5 years of age (n=95) | | | |
| Cfa6.6 | -0.328 (0.510) | -0.884 (0.157) | -0.526 (0.217) |
| Cfa6.7 | 0.202 (0.686) | 0.910 (0.146) | 0.077 (0.856) |
| Cfa6.66 | -0.704 (0.160) | -0.941 (0.136) | -0.273 (0.545) |
| Cfa6.83 | 0.524 (0.296) | 0.528 (0.403) | 0.006 (0.990) |
| All dogs owned from 1-5 years (n=115) | | | |
| Cfa6.6 | -0.210 (0.474) | -1.056 (0.057) | 0.473 (0.454) |
| Cfa6.7 | -0.020 (0.944) | 0.736 (0.183) | -0.686 (0.274) |
| Cfa6.66 | **0.656 (0.038)** | **1.184 (0.037)** | 0.684 (0.292) |
| Cfa6.83 | 0.152 (0.629) | 0.660 (0.243) | -0.215 (0.739) |
| Pet dogs owned from 1-5 years (n=65) | | | |
| Cfa6.6 | 0.286 (0.426) | -0.138 (0.692) | 0.492 (0.131) |
| Cfa6.7 | 0.428 (0.233) | **0.736 (0.026)** | 0.006 (0.984) |
| Cfa6.66 | 0.220 (0.578) | 0.254 (0.517) | 0.092 (0.803) |
| Cfa6.83 | 0.170 (0.668) | 0.301 (0.441) | -0.001 (0.997) |
| Pet dogs owned >5 years (n=96) | | | |
| Cfa6.6 | -0.019 (0.966) | -0.366 (0.580) | 0.058 (0.804) |
| Cfa6.7 | 0.503 (0.275) | **1.355 (0.040)** | -0.071 (0.761) |
| Cfa6.66 | -0.541 (0.250) | -0.664 (0.318) | -0.197 (0.415) |
| Cfa6.83 | 0.397 (0.400) | 0.361 (0.588) | 0.192 (0.431) |

**Supplementary Table S10.** Differences in scores between assistance dogs and pet dogs on the C-BARQ© with respect to the defined behavioral axes. Bolded values indicate *p*<0.05. (Abbreviations: CI, confidence interval).

|  |  | **Median Scores** | | **Mean Score (+/-95% CI)** | |
| --- | --- | --- | --- | --- | --- |
| **Behavioral Summary** | ***P-*Value^τ^** | **Assistance** | **Pet** | **Assistance** | **Pet** |
| Excitability | 0.83 | 1.5 | 2 | 1.9 (0.17) | 2.1 (0.20) |
| **Stranger Directed Aggression** | **5.5x10^-7^** | **0** | **0.67** | **0.17 (0.11)** | **0.73 (0.16)** |
| Stranger Directed Fear | 1 | 0 | 0 | 0.52 (0.24) | 0.55 (0.17) |
| Nonsocial fear | 1 | 1 | 0.67 | 1.1 (0.13) | 0.98 (0.17) |
| **Dog directed fear** | **9.1x10^-4^** | **0** | **0.5** | **0.40 (0.14)** | **0.87 (0.14)** |
| Touch sensitivity | 1 | 1 | 1 | 1.1 (0.08) | 1.2 (0.22) |
| Separation related problems | 0.19 | 0.84 | 0.33 | 1.1 (0.24) | 0.63 (0.16) |
| Attachment attention seeking | 1 | 3 | 3 | 2.9 (0.17) | 2.7 (0.17) |
| Training difficulty | 0.41 | 1.7 | 1.7 | 1.5 (0.15) | 1.8 (0.14) |
| Chasing | 0.05 | 2 | 3 | 2.2 (0.19) | 2.6 (0.27) |
| **Energy** | **0.03** | **2** | **2.5** | **1.8 (0.21)** | **2.3 (0.17)** |
| Owner Directed Aggression | 0.92 | 0 | 0 | 0.01 (0.02) | 0.06 (0.04) |

τ Bonferroni corrected P-values are obtained using Mann-Whitney U tests, testing whether the distributions of behavioral summary scores are significantly different across Assistance and pet dogs.


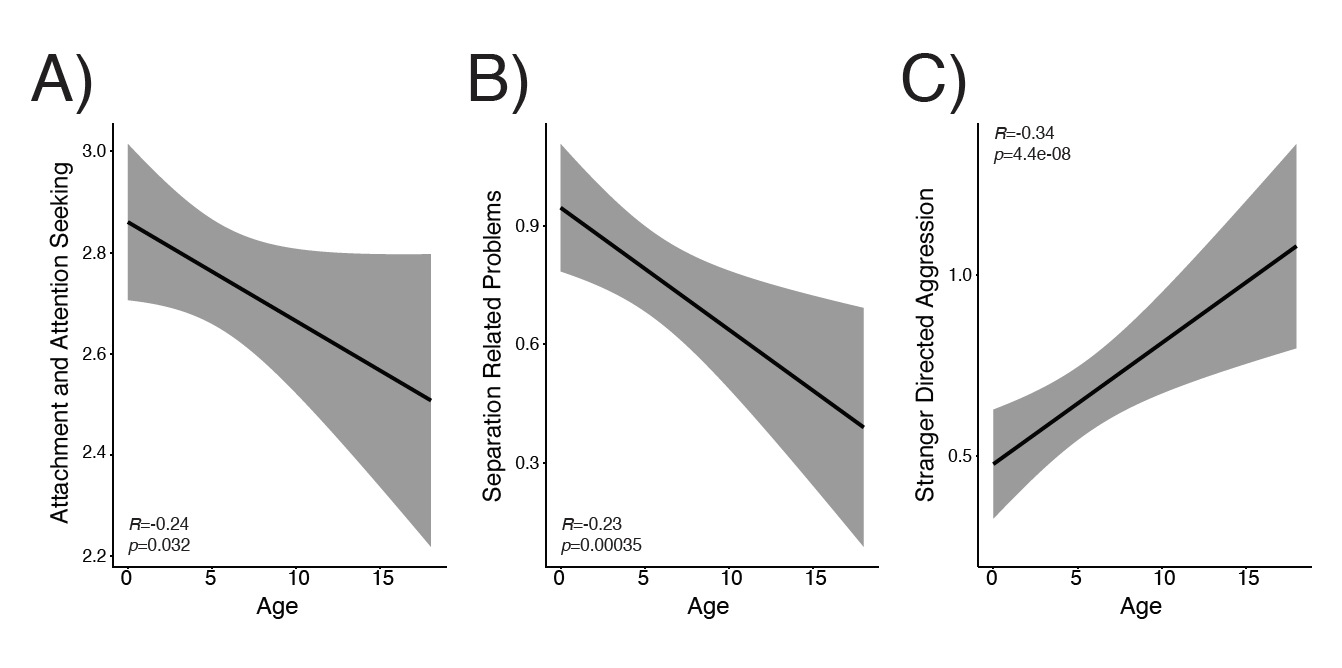


Supplementary Figure S1. Correlation between age and C-BARQ© A) Attachment/attention seeking; B) Separation-related problems; and C) Stranger-directed aggression.

**Supplementary Figure S2**: Frequency of the inserted allele in Retriever-clade assistance (n=147) and pet dogs (n=58) at each locus **A)** Cfa6.6; **B)** Cfa6.7; **C)** Cfa6.66; and **D)** Cfa6.83.

**
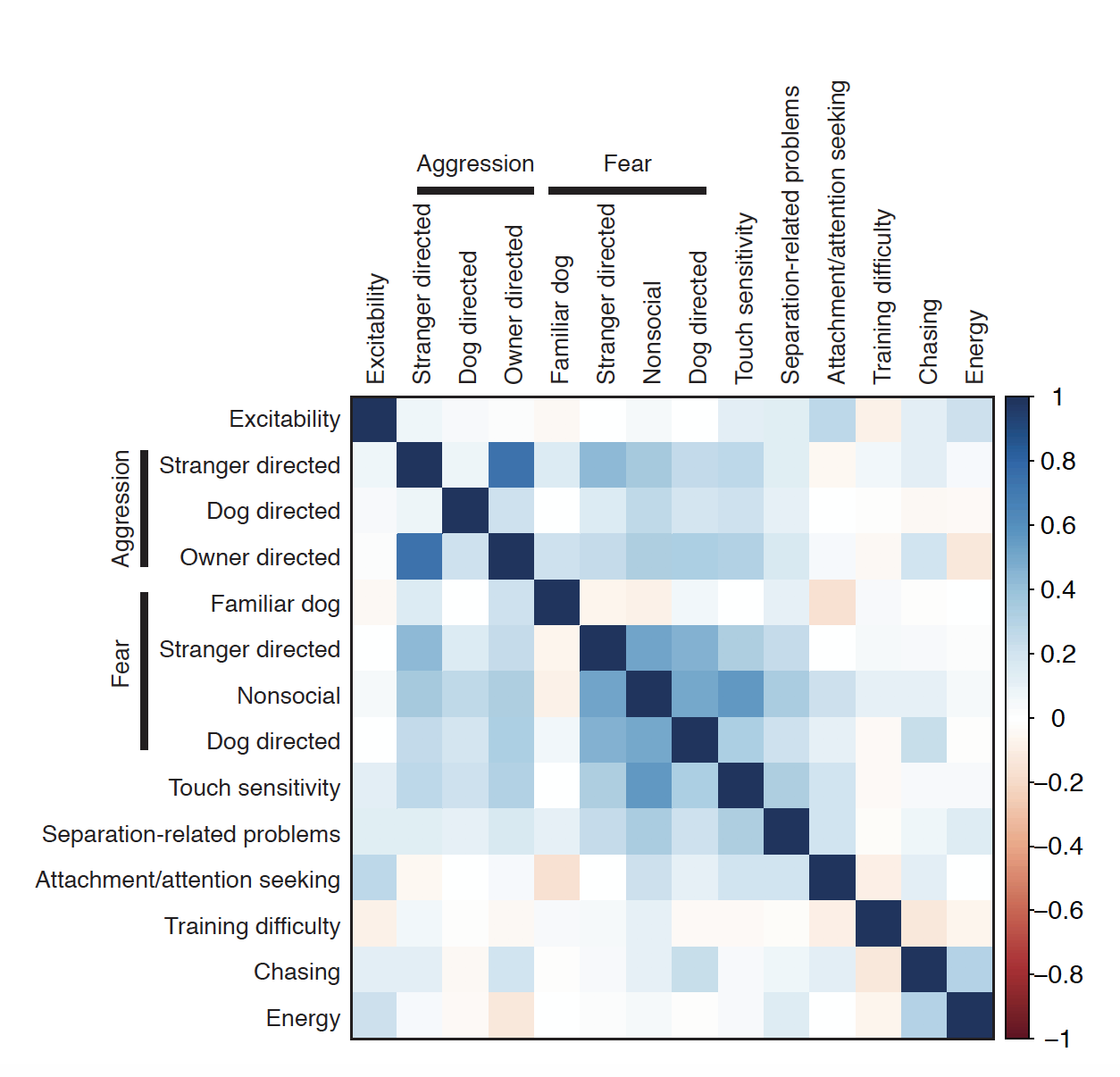
**

Supplementary Figure S3. Pairwise correlations between the 14 behavioral axes of the C-BARQ©.
